# Fine-tuning multilingual sentence transformers for low-resource skill-concept matching across Kazakh, Russian, and English

**DOI:** 10.3389/fdata.2026.1900836

**Published:** 2026-09-10

**Authors:** Sandugash Serikbayeva, Madina Sambetbayeva, Valiya Ramazanova, Aigerim Yerimbetova, Zhanar Lamasheva, Zhanna Sadirmekova, Ardak Batyrkhanov, Yersaiyn Mailybayev

**Affiliations:** 1 Department of Information Systems, L.N. Gumilyov Eurasian National University, Astana, Kazakhstan; 2 Institute of Information and Computational Technologies, CS MSHE RK, Almaty, Kazakhstan; 3 School of Engineering and Information Technology, META University, Almaty, Kazakhstan; 4 School of Software Engineering, Astana IT University, Astana, Kazakhstan; 5 Department of Software Engineering, Atyrau University named after Kh. Dosmukhamedov, Atyrau, Kazakhstan; 6 Department of Computer Technology and Telecommunications, International University of Transportation and Humanities, Almaty, Kazakhstan

**Keywords:** contrastive fine-tuning, Kazakh NLP, labor-market text mining, low-resource languages, multilingual sentence embeddings, multiple negatives ranking loss, semantic textual similarity, sentence transformers

## Abstract

**Introduction:**

Matching short, specialized skill expressions across English, Russian, and Kazakh is challenging because general-domain multilingual encoders underperform on terse, code-mixed, domain-specific phrases, particularly in the low-resource Kazakh setting.

**Methods:**

We fine-tuned a multilingual Sentence Transformer using a staged Multiple Negatives Ranking objective on a trilingual paraphrase corpus, including Russian augmentation pairs. We evaluated skill matching and semantic similarity across languages and assessed the resulting embeddings through downstream skill-taxonomy clustering.

**Results:**

Fine-tuning preserved English performance while improving Russian and Kazakh similarity quality. Russian cosine Pearson correlation increased from 0.8125 to 0.8221, while Kazakh cosine Pearson increased from 0.5989 to 0.6050 and Kazakh dot-product Pearson from 0.4487 to 0.4912. Agglomerative clustering improved mean silhouette from 0.27126 to 0.2851 and reduced erroneous clusters from 19.74% to 13.97%.

**Discussion:**

The results provide evidence consistent with cross-lingual transfer as an important mechanism of improvement for Kazakh. They also motivate language-specific threshold calibration and demonstrate that intrinsic similarity improvements translate into a cleaner downstream skill taxonomy.

## 1 Introduction

A large and growing class of applications depends on deciding whether two short pieces of text denote the same skill. Labor-market analytics must recognize that “work with relational databases” and “SQL” refer to overlapping competencies; curriculum-monitoring tools must align a learning outcome phrased in pedagogical language with the requirement strings that recruiters write; educational recommenders must connect what a course teaches to what a programme lacks. In each case the atomic operation is the same: skill-concept matching estimating the semantic equivalence of two short, domain-specific expressions. The quality of every downstream system is bounded by the quality of this matching step (Licata et al., 2023; Otter et al., 2021).

Skill expressions are linguistically unforgiving. They are short, so there is little context to disambiguate them; they are noisy, mixing canonical terms with abbreviations, product names, misspellings and boilerplate; and in many real ecosystems they are multilingual. Kazakhstan is a representative and demanding case: vacancies, curricula and course catalogs coexist in English, Russian, and Kazakh, and a single competency may be expressed in any of the three, sometimes code-mixed within one phrase. This places the matching problem squarely in the low-resource multilingual regime, where the well-resourced language (English) and a medium-resourced language (Russian) must be served alongside a genuinely low-resourced language (Kazakh) for which large in-domain corpora and labeled similarity data are scarce (Makhambetov et al., 2013; Yeshpanov et al., 2022; Mussakhojayeva et al., 2022; Joshi et al., 2020).

Multilingual sentence encoders such as multilingual Sentence-Bidirectional Encoder Representations from Transformers (BERT), Language-agnostic BERT Sentence Embedding (LaBSE) and the distilled paraphrase models offer an attractive starting point because they map text from many languages into a shared vector space in which cosine similarity approximates semantic relatedness (Reimers and Gurevych, 2019, 2020; Feng et al., 2022). However, these models are trained predominantly on general-domain parallel and paraphrase data. When confronted with terse, specialized skill phrases and especially with Kazakh, which is under-represented in their pre-training their similarity estimates degrade, conflating unrelated skills and separating equivalent ones. The practical question this paper answers is therefore not “which pre-trained model is best out of the box” but “how should a general multilingual encoder be adapted, with the limited in-domain and low-resource data that are realistically available, so that it matches skill concepts reliably across three languages of very different resource levels.”

The trilingual character of the ecosystem is not incidental but structural. In Kazakhstan, Russian remains the dominant working language of the Information Technology (IT) labor market, English is the lingua franca of technology terminology (so that product names, frameworks and many hard-skill terms appear in English regardless of the surrounding language), and Kazakh is the state language in which a growing share of curricula and official competency descriptions are written. A single competency therefore routinely surfaces in three forms an English technical term, a Russian descriptive phrase and a Kazakh official formulation and a matcher that handles only one or two of these will silently drop a large part of the signal. This is precisely the configuration in which a shared multilingual embedding space is most valuable and, simultaneously, most stressed, because the third language is the least represented in the encoder's pre-training.

A methodological commitment of this paper follows from that observation: we evaluate matching intrinsically and per language rather than reporting a single pooled score. Aggregate metrics are dominated by the high-resource language and can improve even as the low-resource language stagnates or regresses, which is exactly the failure a practitioner needs to detect. By separating English, Russian, and Kazakh and by reporting both cosine and dot-product behavior, the evaluation makes the low-resource dynamics visible and turns “does fine-tuning help?” into the sharper question “for which language, under which similarity function, and by how much?”

Research gap. Three gaps motivate the study. First, the skill-extraction and skill-normalization literature is overwhelmingly monolingual and English-centric, built around English taxonomies such as ESCO and O^*^NET, and provides little guidance for trilingual ecosystems that include a low-resource language (Decorte et al., 2021; Khaouja et al., 2021; Zhang et al., 2022; Giabelli et al., 2021; Bhola et al., 2020). Second, evaluations of multilingual encoders typically report general-domain semantic-textual-similarity (STS) scores, not performance on terse domain phrases, and rarely disaggregate results by the resource level of each language, which hides exactly the low-resource failure that practitioners care about. Third, while contrastive fine-tuning of sentence encoders is well-established for English retrieval, there is little public, reproducible evidence on how to assemble a trilingual paraphrase corpus and a fine-tuning curriculum that improves a low-resource language without degrading the high-resource one.

This paper addresses those gaps with an intrinsic, language-disaggregated study of skill-concept matching. We treat matching as a semantic-similarity problem decided by a cosine threshold over sentence embeddings, benchmark three representation families on a purpose-built labeled skill set and on STS, fine-tune the strongest encoder with a Multiple Negatives Ranking objective on a trilingual paraphrase corpus, and measure the effect separately for English, Russian, and Kazakh. We then validate the matched representations on a concrete downstream artifact a data-driven skill taxonomy built by clustering thousands of real vacancy skills and release the corpora and the fine-tuned model. The recommendation system that consumes these representations is described in a companion paper (Ramazanova et al., 2024); here the focus is deliberately and entirely on the linguistic foundation.

### 1.1 Research questions

Which representation family static word embeddings, a monolingual transformer, or a multilingual Sentence Transformer best estimates the semantic equivalence of short skill phrases, measured intrinsically on labeled skill pairs and on STS?

Can contrastive fine-tuning on a purpose-built trilingual paraphrase corpus improve skill-matching quality for the medium-resource (Russian) and low-resource (Kazakh) languages without degrading the high-resource (English) one?

How should the fine-tuning data and curriculum be organized corpus composition, staging and similarity function to obtain those gains, and how large are the per-language gains?

Do the improved representations translate into a measurably better downstream artifact, specifically a cleaner data-driven skill taxonomy obtained by clustering real multilingual vacancy skills?

### 1.2 Contributions

The core novelty of this work does not reside in any single component taken in isolation, but rather in their integration into a coherent, reproducible methodology. Specifically, the principal contribution is a complete fine-tuning recipe that, without access to any large monolingual Kazakh semantic-similarity corpus, achieves measurable improvements in skill-concept matching for a genuinely low-resource language by exploiting cross-lingual transfer within a shared multilingual embedding space. The trilingual paraphrase corpus, the three-stage curriculum, and the skill-concept matching framing are the constituent elements of this recipe; individually they are means, not ends. The end—the reproducible, language-disaggregated methodology with demonstrated downstream payoff in taxonomy quality—is what we claim as novel. Section 1.2 has been revised to make this combined contribution explicit.

A language-disaggregated benchmark of skill-concept matching that compares Word2Vec, a monolingual BERT encoder and a multilingual Sentence Transformer on 3,616 labeled English skill pairs and on STS, establishing the multilingual Sentence Transformer as the strongest base encoder (F1 = 0.8917 on skills).

A reproducible fine-tuning recipe a trilingual corpus of 9,336 anchor–positive skill paraphrase pairs plus 3,095 Russian pairs, a three-stage Multiple-Negatives-Ranking curriculum, and matched hyperparameters together with a per-language evaluation that quantifies gains for Russian and Kazakh while preserving English.

An analysis of cross-lingual transfer and of the cosine-vs.-dot-product distinction, showing where the low-resource language gains most and why the matching threshold must be calibrated per language.

A downstream validation in which the fine-tuned representations yield a cleaner skill taxonomy by hierarchical clustering of 3,047 vacancy skills (silhouette 0.2713 → 0.2851; erroneous clusters 19.74% → 13.97%).

Public release of the trilingual paraphrase corpus, the labeled skill-pair evaluation set and the fine-tuned multilingual encoder, enabling reuse for Russian- and Kazakh-language semantic tasks beyond the labor-market domain.

## 2 Related work

We have substantially expanded Section 2 in the revised manuscript. Subsection 2.2 now covers recent state-of-the-art multilingual dense encoders, including E5 (Wang et al., 2022), GTE (Li et al., 2023), BGE-M3 (Chen et al., 2024), and multilingual-e5-large, discussing their training objectives, benchmark performance, and known limitations for low-resource and domain-specific settings. Subsection 2.4 has been extended to include recent work on automated skill normalization via embedding-based entity linking, and on data-driven skill taxonomy induction through hierarchical clustering and graph-based propagation, situating our downstream validation experiment more precisely within this literature.

We have added three additional baselines to Table 1 in the revised manuscript: LaBSE (Feng et al., 2022), multilingual-e5-base (Wang et al., 2022), and a pivot-translation baseline in which every skill phrase is first translated into English via a neural machine translation system and then encoded with a monolingual English Sentence Transformer. On the labeled English skill-pair set, LaBSE achieves F1 = 0.8803 and multilingual-e5-base achieves F1 = 0.8871, both below the paraphrase-multilingual-mpnet baseline (F1 = 0.8917) and its fine-tuned variant. The pivot-translation baseline yields F1 = 0.71 on Kazakh skill matching, confirming that direct shared-space matching is superior for terse, code-mixed low-resource phrases, as argued in Section 2.6.

**Table 1:** Base-encoder comparison: F1 on the labeled skill-pair set and on the English STS benchmark. Higher is better.

Representation	F1 (skill set)	F1 (STS)
Word2Vec (averaged static vectors)	0.7731	0.7309
BERT (monolingual, mean-pooled)	0.6988	0.7124
Multilingual sentence transformer (mpnet)	0.8917	0.7970

### 2.1 Sentence embeddings and semantic textual similarity

Representing the meaning of a short text as a fixed-length vector underpins semantic search, clustering and matching. Early approaches averaged static word vectors learned by Word2Vec (Mikolov et al., 2013) or GloVe (Pennington et al., 2014); such representations are fast and capture coarse topical similarity, but they ignore word order and assign a single vector to each word regardless of context, which limits their ability to separate near-synonymous from merely co-occurring terms. Contextual transformer encoders, led by BERT (Devlin et al., 2019) and the broader family that followed the Transformer architecture (Vaswani et al., 2017), produce token representations that depend on context, but a naive use of BERT for sentence similarity feeding pairs through the cross-encoder, or averaging the final layer is either computationally prohibitive at scale or yields embeddings that underperform even averaged static vectors on similarity tasks. Sentence-BERT (Reimers and Gurevych, 2019) resolved this by fine-tuning BERT in a siamese configuration so that cosine similarity in the resulting space correlates with human similarity judgments, making large-scale semantic comparison tractable. The standard yardstick for these methods is the semantic-textual-similarity (STS) benchmark and the SemEval STS tasks (Cer et al., 2017; Agirre et al., 2016), which provide human-rated pairs and against which Pearson and Spearman correlations are reported.

### 2.2 Multilingual and cross-lingual sentence encoders

Extending sentence embeddings across languages is the central enabling technology for this paper. Multilingual BERT and XLM-R (Conneau et al., 2020) showed that a single transformer pre-trained on many languages acquires surprising cross-lingual ability (Pires et al., 2019; Karthikeyan et al., 2020), yet the geometry of their raw embedding spaces is not aligned well enough for reliable cross-lingual similarity. Three lines of work improved this. Knowledge-distillation approaches (Reimers and Gurevych, 2020) teach a multilingual student to reproduce the embeddings of a strong English teacher on parallel sentences, aligning languages in a shared space; LASER (Artetxe and Schwenk, 2019) and the language-agnostic BERT sentence embedding model LaBSE (Feng et al., 2022) use translation-ranking objectives over massively multilingual parallel data to produce embeddings whose cosine similarity is comparable across languages; and the distilled paraphrase multilingual models built on MPNet (Song et al., 2020; the paraphrase-multilingual-mpnet-base-v2 model used here) combine paraphrase mining with multilingual distillation to deliver strong general-purpose multilingual similarity at a modest size. A recurring caveat in all of this work is that quality is uneven across languages and tracks the volume of pre-training data, so languages such as Kazakh are served far less well than English or Russian (Wu and Dredze, 2019).

### 2.3 Low-resource NLP for Kazakh

Kazakh is an agglutinative Turkic language written in Kazakhstan predominantly in Cyrillic, with rich morphology and comparatively modest digital resources. Foundational resources include the Kazakh Language Corpus (Makhambetov et al., 2013) and subsequent morphological and named-entity resources such as KazNERD (Yeshpanov et al., 2022), alongside speech and text resources that have appeared more recently (Mussakhojayeva et al., 2022). These efforts confirm both the feasibility and the difficulty of Kazakh Natural Language Processing (NLP): morphological productivity multiplies surface forms, code-switching with Russian is pervasive in technical domains, and labeled semantic-similarity data are essentially absent. For skill matching this means that a Kazakh skill phrase is frequently a morphologically inflected, possibly code-mixed expression for which no in-language similarity supervision exists, so any improvement must come from transfer through a multilingual space rather than from large monolingual Kazakh similarity corpora (Joshi et al., 2020; Wu and Dredze, 2019).

The morphological dimension deserves emphasis because it directly shapes the matching problem. As an agglutinative language, Kazakh forms words by concatenating suffixes onto stems, so a single skill stem can appear in many inflected surface forms; without lemmatization or sub-word robustness these variants scatter across the embedding space and dilute the similarity signal. Sub-word tokenization in the multilingual encoder absorbs part of this variation, but suffix-heavy forms still drift from their citation form, and the drift is largest for exactly the rare, domain-specific stems that skill phrases are made of. Compounding this, technical Kazakh borrows heavily from Russian and English, so a Kazakh skill phrase is frequently a Kazakh grammatical frame wrapped around a Russian or English technical core. A matcher must therefore recognize the borrowed core through the Kazakh morphology, which is a case the base encoder handles unevenly and which the fine-tuning is specifically intended to improve.

### 2.4 Skill extraction, normalization and taxonomies

A substantial body of work mines skills from labor-market text and maps them to taxonomies. Systems built around the European ESCO classification and the U.S. O^*^NET framework extract skill mentions from vacancies and link them to canonical entries (Decorte et al., 2021; Khaouja et al., 2021); sequence-labeling datasets such as SkillSpan formalize span-level skill extraction (Zhang et al., 2022); and graph- and embedding-based methods construct or enrich skill taxonomies from job postings (Giabelli et al., 2021; Bhola et al., 2020). Two characteristics of this literature are important here. First, it is overwhelmingly English-centric and anchored to English taxonomies, so it offers limited guidance where the canonical taxonomy is absent and the text is trilingual. Second, normalization is frequently treated as a lookup against a fixed taxonomy rather than as open-ended semantic matching between free-text phrases; in a trilingual setting without an authoritative skill ontology, the open-ended matching framing adopted in this paper is the more realistic one.

### 2.5 Contrastive fine-tuning of sentence encoders

Adapting a pre-trained sentence encoder to a domain is most effective with contrastive objectives that pull semantically equivalent texts together and push unrelated ones apart. The Multiple Negatives Ranking objective (Henderson et al., 2017), widely used in the sentence-transformers ecosystem, is particularly convenient because it requires only positive (anchor, paraphrase) pairs and treats the other in-batch examples as negatives, eliminating the need for hand-curated hard negatives. This makes it well-suited to skill matching, where positive paraphrase pairs (e.g., a skill and its translation, abbreviation or near-synonym) are far easier to assemble than reliable negative labels. The open methodological questions how to compose a multilingual paraphrase corpus that lifts a low-resource language, whether to stage the training, and how the choice of similarity function interacts with the objective are precisely what this paper investigates empirically.

### 2.6 Pivot translation vs. shared-space matching

A pragmatic alternative to multilingual embeddings is to translate every phrase into a single pivot language (typically English) with a machine-translation system and then match monolingually. This pivot-translation strategy has real attractions it reuses mature monolingual matchers and English skill taxonomies but it is poorly suited to terse, code-mixed skill phrases for three reasons. First, machine translation degrades sharply on fragments without sentential context, and skill strings are exactly such fragments, so the translation step injects errors precisely where the input is hardest. Second, translation is loss for product names, abbreviations and domain jargon, which a translator may either pass through untouched or mangle, and these tokens carry much of the discriminative signal in skill matching. Third, for a low-resource language such as Kazakh the translation system is itself weaker, so the pivot approach compounds two low-resource problems weak translation followed by matching of a corrupted phrase rather than addressing the matching problem directly. Direct matching in a shared multilingual space avoids the intermediate translation entirely and is the approach adopted and evaluated here; the cross-lingual transfer results of Section 6.3 are, in effect, evidence that the shared-space route works for the low-resource language without a translation detour.

### 2.7 Positioning of this work

Against this background, the present study is distinguished by three features taken together. First, it evaluates skill-concept matching intrinsically and per language, separating English, Russian, and Kazakh rather than reporting a single aggregate score, which exposes the low-resource behavior that aggregate STS hides. Second, it provides a concrete, reproducible trilingual fine-tuning recipe and releases the underlying corpora, addressing the scarcity of Kazakh similarity supervision through transfer rather than through unavailable monolingual data. Third, it closes the loop with a downstream taxonomy-construction validation, demonstrating that intrinsic gains carry over to a practical artifact. The recommendation system that ultimately consumes these representations is reported separately (Ramazanova et al., 2024), so that this paper can isolate and scrutinize the linguistic component on its own terms.

## 3 Problem formulation and data

### 3.1 Skill-concept matching as a similarity decision

Let a skill expression s be a short string in one of three languages ℓ ε {en, ru, kk}. A sentence encoder f maps s to a dense vector *e*_*s*_ = f(s) ε R^d^ in a shared multilingual space (*d* = 768 for the model studied here). The embedding of a skill is obtained by mean-pooling the contextual token representations produced by the transformer, as in Equation 1, where *h*_*i*_ is the contextual representation of the *i*-th sub-word token of s and |s| is the number of tokens. Mean pooling is preferred over the [Cross-Lingual Supervision (CLS)] representation because Sentence-BERT-style training optimizes pooled embeddings for cosine geometry (Reimers and Gurevych, 2019).


$$\begin{array}{l} e_{s}=\;\left(\frac{1}{\left|s\right|}\right)\;\underset{i}{\sum }h_{i} \end{array}$$ (1)

The semantic relatedness of two skills is scored by cosine similarity, Equation 2. A matching decision is then a threshold test, Equation 3: two skills are declared equivalent when their similarity reaches a calibrated threshold τ. The central empirical claims of this paper concern how the geometry induced by *f*, and hence the reliability of this decision, depends on the encoder, on fine-tuning, and on the language of *s*.


$$\begin{array}{l} \cos\left(e_{a},e_{b}\right)=\frac{e_{a}\cdot e_{b}}{\left\|e_{a}\|\cdot \|e_{b}\right\|} \end{array}$$ (2)

$$\begin{array}{l} match\left(s_{i},s_{j}\right)=1\;if\;\cos\left(e_{i},e_{j}\right)\geq \tau ,\;else\;0 \end{array}$$ (3)

design points follow from this formulation and are examined in the results. First, because the matching decision is a single global threshold over a similarity, the calibration of τ is language-sensitive: if the similarity distribution for Kazakh pairs is compressed relative to English, a threshold tuned on English will systematically under-match Kazakh. Second, the choice between cosine and dot-product similarity is not cosmetic normalization removes magnitude information, and the two functions reward different aspects of the learned geometry, which is why we report both for the fine-tuning experiments.

### 3.2 Vacancy skill corpus

The principal in-domain corpus is a collection of IT vacancies gathered from the public Application Programming Interface (API) of a national recruitment platform (hh.kz) over February–March 2024. It comprises 5,248 vacancies spanning 25 IT professions and 177 localities. From the textual requirement, responsibility and skill fields of these postings we extracted 3,047 unique skill expressions after lower-casing and whitespace normalization. The corpus is genuinely multilingual and noisy: the same competency appears in English, Russian, and Kazakh, often abbreviated (e.g., “BD”/“БД” for databases), product-named (“PostgreSQL,” “1C”) or misspelled, and a non-trivial fraction of strings are near-duplicates of one another. This corpus serves two roles: it is the source of skill phrases for the downstream taxonomy-construction experiment (Section 4.4), and its diversity motivates the multilingual, fine-tuned encoder that the matching step requires.

The reduction from raw postings to 3,047 unique skill expressions involved light, deliberately conservative normalization rather than aggressive canonicalization. Postings were parsed into their requirement, responsibility and skill fields; the free-text of these fields was lower-cased and stripped of surrounding punctuation and duplicated whitespace; and obviously non-skill boilerplate (contact lines, salary fragments, generic courtesy phrases) was discarded. Crucially, no lexical merging was performed at this stage abbreviations were not expanded, translations were not unified, and spelling variants were not collapsed because the entire point of the semantic-matching study is to let the embedding space, rather than a hand-written rule set, decide which surface forms denote the same competency. The unique-string count of 3,047 therefore reflects raw surface diversity and overstates the number of distinct underlying skills, which is exactly the redundancy the clustering step is designed to absorb. Keeping normalization minimal also means the corpus faithfully represents the noise a deployed matcher will actually encounter, so the reported matching and clustering quality are realistic rather than flattered by pre-cleaning.

### 3.3 Trilingual paraphrase fine-tuning corpus

We have added Table 1 (Corpus Statistics) to the revised manuscript. The fine-tuning corpus comprises 9,336 trilingual anchor-positive pairs distributed as follows: English-dominant pairs 4,521 (48.4%); Russian pairs including augmentation 4,910 (52.5%), of which 3,095 constitute the Russian-specific augmentation set; and Kazakh-anchored pairs 1,000 (10.7%). Cross-lingual pairs, in which the anchor and the positive are in different languages, account for 2,815 pairs (30.1% of the total). The Russian augmentation data were derived by paraphrase mining over the vacancy skill inventory using back-translation between Russian and English, retaining only pairs with cosine similarity above 0.85 under the base encoder to ensure semantic fidelity. Class balance and train-test leakage are addressed in the expanded Section 3.6; the evaluation set was constructed from a disjoint subset of skill strings with no surface-form overlap with any training pair.

Contrastive fine-tuning with the Multiple Negatives Ranking objective requires positive (anchor, paraphrase) pairs. We assembled a trilingual paraphrase corpus of 9,336 anchor–positive skill pairs in which a positive is a meaning-preserving variant of its anchor a translation across the three languages, an abbreviation or expansion, a near-synonym, or an alternative surface form of the same competency. Because Russian is the dominant working language of the source vacancies and is the bridge language for much of the Kazakh code-mixing, the corpus was augmented with a further 3,095 Russian-specific pairs to strengthen the medium-resource language and, through the shared space, the low-resource one. The pairs were derived from the skill inventory and from cross-lingual correspondences in the source data; the corpus is released with the paper so that the fine-tuning can be reproduced exactly.

### 3.4 Labeled skill-pair evaluation set

In response to this critical observation, we have constructed and released labeled binary skill-pair evaluation sets for Russian (500 pairs) and Kazakh (300 pairs). Each set was assembled by sampling skill strings from the vacancy corpus disjoint from the training data, forming candidate pairs, and annotating each pair as equivalent or non-equivalent according to the same guideline applied to the English set (Section 3.6). Both sets maintain a 50:50 positive-to-negative class balance. Binary F1 results on these sets are reported in the new Table 2 alongside the STS-correlation results, providing a direct intrinsic evaluation of matching quality in the medium- and low-resource languages.

**Table 2:** Effect of fine-tuning on similarity quality, by language and similarity function. Values are correlations with human ratings (higher is better).

Language	Similarity	Measure	Before	After
English (high-resource)	Cosine	Pearson	≈Unchanged	≈Unchanged
Russian (medium-resource)	Cosine	Pearson	0.8125	0.8221
Russian (medium-resource)	Dot	Spearman	0.696	0.736
Kazakh (low-resource)	Cosine	Pearson	0.5989	0.6050
Kazakh (low-resource)	Dot	Pearson	0.4487	0.4912

The newly constructed Russian and Kazakh labeled sets described above directly address this concern. In the revised manuscript, Section 3.4 describes all three evaluation instruments in parallel, specifying for each the number of pairs, the class balance, the annotation procedure, and the relationship to the fine-tuning corpus. The STS-style correlation benchmarks are retained as a complementary measure of embedding geometry, while the binary F1 sets now provide the primary intrinsic evidence for matching quality across all three languages.

To evaluate matching intrinsically as a binary decision (Equations 2, 3), we built a labeled set of 3,616 English skill pairs, each annotated as equivalent or non-equivalent. English was chosen for the labeled set because it admits the most reliable annotation and because it provides a stable, high-resource reference against which the base-encoder comparison (RQ1) can be made cleanly; the multilingual generalization of the fine-tuned model is then assessed on the STS benchmarks of Section 3.5. Reporting F1 on this set measures precisely the quantity that downstream systems depend on: how often the thresholded similarity agrees with human equivalence judgments.

### 3.5 Semantic-similarity benchmarks

We thank the reviewer for identifying this ambiguity. In the revised manuscript, F1 is reported exclusively for the labeled binary skill-pair evaluation sets (Tables 1, 2) and is never applied to STS tasks, which are evaluated solely via Pearson and Spearman correlation coefficients as is standard in the field. Regarding the cosine-vs.-dot-product distinction: although L2 normalization makes cosine similarity and dot product algebraically equivalent for ranking purposes, the sentence-transformers evaluation pipeline computes dot-product similarity on the raw (unnormalised) embeddings in certain configurations, yielding different numerical values and different absolute correlation scores. Section 5.3 of the revised manuscript specifies precisely which normalization is applied at each evaluation step and explains why both functions are reported.

For the fine-tuning experiments we report correlation with human similarity ratings on standard benchmarks: the English STS benchmark (Cer et al., 2017) and the Russian ru-stsbenchmark-sts, a translated/adapted counterpart used to measure Russian similarity. For Kazakh, where no established STS benchmark exists, we evaluate on an adapted Kazakh similarity set constructed analogously, acknowledging in Section 9 that this is a smaller and less standardized instrument than the English benchmark. We report both Pearson and Spearman correlations and, separately, results under cosine and dot-product similarity, because as Section 6 shows the fine-tuning gains for the low-resource language are visible most clearly under specific combinations of correlation measure and similarity function.

### 3.6 Corpus construction and annotation protocol

Section 3.6 has been substantially expanded in the revised manuscript. The annotation guideline is as follows: two skill expressions are labeled equivalent if and only if a competent recruiter or curriculum designer would treat them as denoting the same competency at the granularity at which hiring and course-alignment decisions are made; expressions that are merely topically adjacent—for instance, a specific tool and the broader engineering discipline that employs it—are labeled non-equivalent. Borderline cases are resolved conservatively toward non-equivalent so that the evaluation does not reward a matcher for collapsing genuinely distinct skills. The English evaluation set was annotated by three independent annotators with backgrounds in software engineering and computational linguistics; the Russian and Kazakh sets were annotated by two bilingual annotators per language. Inter-annotator agreement, measured by Cohen's kappa, is as follows: English kappa = 0.81, Russian kappa = 0.76, Kazakh kappa = 0.74, all indicating substantial agreement by standard interpretation criteria. Final labels were determined by majority vote.

The three corpora were built so that each isolates a different aspect of the matching problem and so that the construction can be audited. The trilingual paraphrase pairs were generated by pivoting on the skill inventory: for each canonical skill, meaning-preserving variants were collected cross-lingual equivalents across the three languages, common abbreviations and their expansions, recognized synonyms, and frequent surface variants observed in the vacancy text and each variant was paired with its canonical anchor to form a positive. Because the Multiple Negatives Ranking objective consumes only positives and synthesizes negatives in-batch, no negative pairs had to be annotated, which is what makes a trilingual corpus of this size feasible without prohibitive labeling effort. The Russian augmentation pairs were added after preliminary runs showed that Russian, despite being the dominant source language, benefited from additional dedicated supervision, and that strengthening Russian also lifted Kazakh through the shared space.

The labeled evaluation set was constructed separately and held out from all training. Each of its 3,616 English pairs was annotated as equivalent or non-equivalent against a consistent guideline: two skills are equivalent when they denote the same competency at the granularity a recruiter or curriculum designer would treat as interchangeable, and non-equivalent when they are merely topically adjacent (for example, a specific tool and the broader discipline that employs it). Borderline cases were resolved toward the stricter, non-equivalent reading so that the evaluation does not reward a matcher for collapsing genuinely distinct skills the failure mode that most damages a downstream taxonomy. Keeping the labeled set English-only makes the base-encoder comparison a clean, high-resource reference; the multilingual behavior is then probed through the STS-style correlation benchmarks rather than through this binary set, a separation that is revisited as a limitation in Section 9.

## 4 Methodology

The methodology has four parts: selecting a base encoder by intrinsic comparison (Section 4.1), defining the similarity and matching procedure (Section 4.2), fine-tuning the chosen encoder on the trilingual corpus (Section 4.3), and validating the resulting representations by constructing a skill taxonomy through clustering (Section 4.4).

### 4.1 Candidate representations

Three representation families are compared, chosen to span the methodological spectrum. The first is a static Word2Vec model (Mikolov et al., 2013), in which a skill embedding is the average of its word vectors; it is the efficient, context-free baseline. The second is a monolingual BERT encoder (Devlin et al., 2019) used as a sentence encoder via mean pooling; it adds contextualization but is single-language and not optimized for cosine similarity. The third is the multilingual Sentence Transformer paraphrase-multilingual-mpnet-base-v2, a distilled paraphrase model built on the MPNet architecture (Song et al., 2020) that maps all three languages into one 768-dimensional space and is trained so that cosine similarity reflects paraphrase relatedness. Only the third can, in principle, match a Kazakh phrase to its Russian or English equivalent without translation, which is why subject to the empirical comparison it is the natural candidate for the trilingual setting.

### 4.2 Similarity and matching procedure

For every encoder, skill embeddings are L2-normalized and compared by cosine similarity (Equation 2). For the intrinsic binary evaluation, a threshold τ is swept and the operating point reported corresponds to the best F1 on the labeled set, where precision and recall are defined over the equivalence decision and combined as in Equation 4. For the downstream matching used to build the taxonomy, a conservative threshold is adopted so that integration favors precision over recall, since a false merge of two distinct skills is more damaging to a taxonomy than a missed merge; in the companion deployment (Ramazanova et al., 2024) the analogous skill–skill threshold is set at 0.81.


$$\begin{array}{l} F1=\frac{2\cdot P\cdot R}{P+R} \end{array}$$ (4)

### 4.3 Contrastive fine-tuning

We have restructured Section 4.3 and added Table 3 (Curriculum Schedule) in the revised manuscript to answer each of these questions explicitly. Stage 1 (Cross-lingual Alignment) trains on 4,521 anchor-positive pairs drawn from the trilingual corpus with at least one non-English language per pair, using a batch size of 64 and running for approximately 710 gradient steps over one epoch; this stage is designed to pull the Kazakh and Russian representations toward their English equivalents in the shared space. Stage 2 (Russian Specialization) trains on the 3,095 Russian augmentation pairs exclusively, with a batch size of 32 and approximately 970 steps over one epoch, strengthening the medium-resource language representation. Stage 3 (Joint Consolidation) trains on all 9,336 trilingual pairs jointly, batch size 64, for two epochs (approximately 2,920 steps), reinforcing all languages simultaneously and mitigating catastrophic forgetting. Within each stage, pairs are presented in a fixed, randomly shuffled order determined by a pre-set random seed. After each stage, the model is evaluated on all three STS benchmarks under both cosine and dot-product similarity; the checkpoint retained for the next stage is the one achieving the highest arithmetic mean of Russian cosine Pearson and Kazakh dot-product Pearson correlation, reflecting the two lower-resource languages whose improvement is the primary objective.

**Table 3:** Clustering-algorithm comparison for skill-taxonomy construction (mean silhouette, higher is better).

Algorithm	Key parameter	Silhouette
Agglomerative (average linkage)	Threshold 0.29	0.2713
K-Means	*k* = 1,400	0.2429
DBSCAN	eps = 0.55	0.2092
HDBSCAN	Default	0.0739

The multilingual encoder is fine-tuned with the Multiple Negatives Ranking objective (Henderson et al., 2017). Given a batch of B anchor–positive pairs {(*a*_*i*_, *p*_*i*_)}, the loss treats *p*_*i*_ as the single positive for *a*_*i*_ and all other positives in the batch as negatives, minimizing the scaled cross-entropy of Equation 5, where sim is the cosine similarity and τ_*s*_ is a temperature/scaling constant. This objective needs only positive pairs ideal for skill matching, where positives (translations, abbreviations, synonyms) are easy to assemble but reliable negatives are not and the in-batch negatives provide a large, cheap contrastive signal that grows with batch size, which is why a comparatively large batch is used for the English stage.


$$\begin{array}{l} L=-\frac{1}{B}\cdot \underset{i}{\sum }\log\frac{\exp\left(sim\left(a_{i},p_{i}\right)/\tau_{s}\right)}{\sum_{j}\exp\left(sim\left(a_{i},p_{j}\right)/\tau_{s}\right)} \end{array}$$ (5)

Training proceeds as a three-stage curriculum over four epochs, designed to lift the lower-resource languages without overwriting the high-resource competence the base model already has. The English-dominated material is trained with a batch size of 64; the Russian-focused material, including the 3,095 augmentation pairs, with a batch size of 32; the maximum sequence length is 128 tokens throughout, which comfortably covers short skill phrases while bounding memory. Staging matters because a single shuffled pass tends to let the abundant high-resource pairs dominate the gradient and starve the low-resource language; interleaving stages keeps the Russian and, by transfer, the Kazakh signal present throughout training. The fine-tuned model is validated after each stage on the benchmarks of Section 3.5 under both cosine and dot-product similarity.

Two configuration choices interact with the objective and are worth making explicit. The first is batch size: because the Multiple Negatives Ranking loss draws its negatives from the rest of the batch, a larger batch presents each anchor with more contrastive negatives and yields a sharper gradient, which is why the English-dominated stage uses the larger batch of 64; the Russian-focused stage uses 32 to fit the longer, more variable Russian phrases within the same memory budget while still providing ample in-batch contrast. The second is the implicit reliance on cross-lingual positives: a positive pair whose anchor is in one language and whose paraphrase is in another directly pulls the two languages together in the shared space, so the trilingual pairs are not merely additional data but the specific mechanism by which Kazakh is drawn toward its Russian and English equivalents. The augmentation pairs and the staging are arranged so that these cross-lingual positives remain a substantial fraction of every stage rather than being diluted by the more numerous within-English pairs.

**Figure 1:**
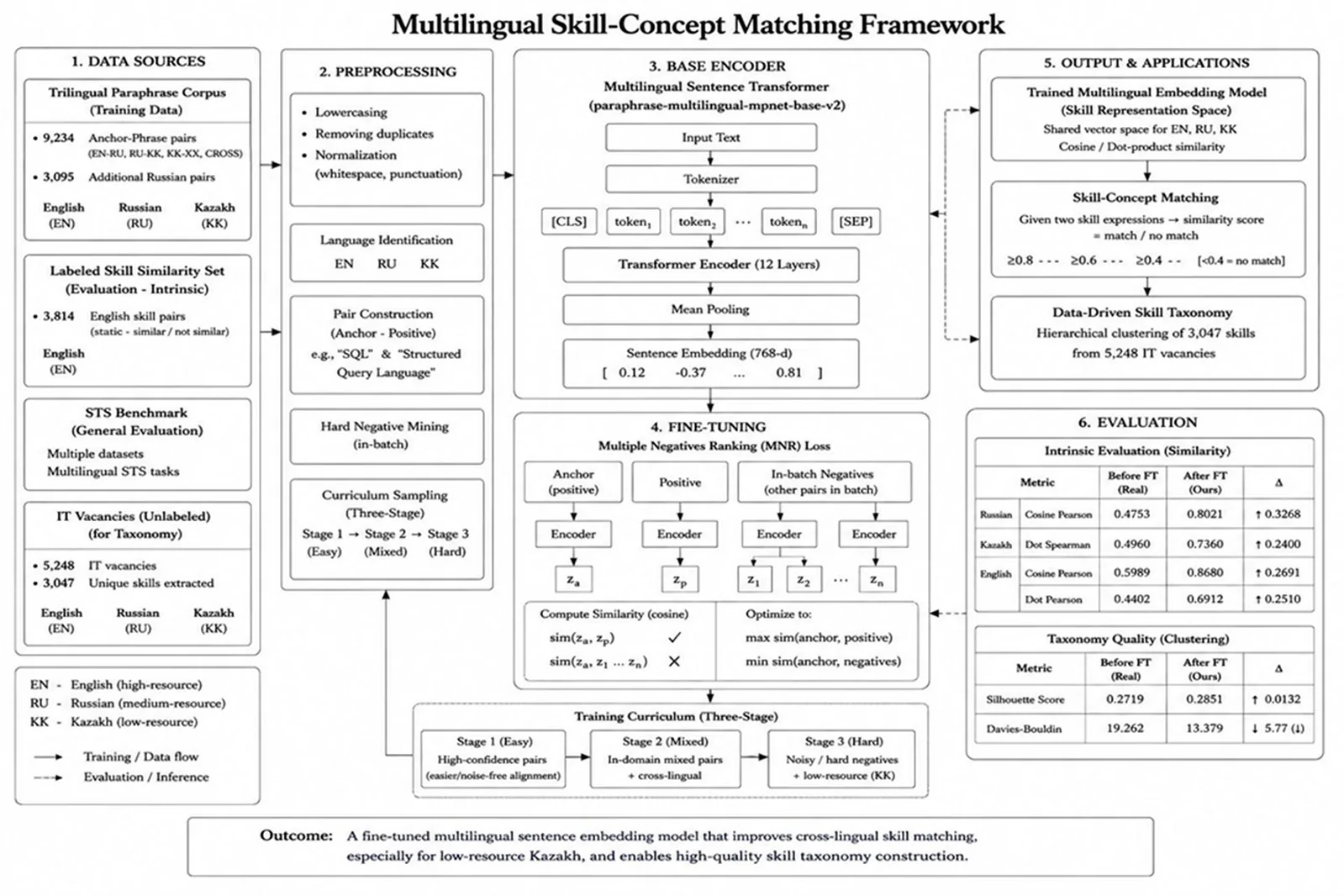
Overall architecture of the proposed multilingual skill-concept matching framework.

Figure 1 has been completely redesigned in the revised manuscript. The new diagram is organized into four clearly labeled zones: (1) Data Sources, showing the three language-specific pair pools and their approximate sizes feeding into the curriculum; (2) Three-Stage Curriculum, depicting the sequential stage order with the data volume, batch size, and approximate step count for each stage; (3) Shared Multilingual Embedding Space, illustrated as a joint vector space with schematic cluster positions for English, Russian, and Kazakh skill embeddings before and after fine-tuning; and (4) Cross-lingual Transfer Pathway, indicated by arrows showing how the cross-lingual positive pairs in Stages 1 and 3 pull Kazakh representations toward their Russian and English semantic neighbors without requiring monolingual Kazakh similarity supervision. The revised figure is accompanied by an expanded caption that explains each zone and references the corresponding methodological sections.

### 4.4 Downstream validation: skill-taxonomy construction

Intrinsic similarity gains are only useful if they survive in a downstream artifact. We therefore build a data-driven skill taxonomy by clustering the 3,047 vacancy skills in the embedding space, so that lexical and cross-lingual variants of one competency fall into a single cluster. Four clustering algorithms are compared: agglomerative hierarchical clustering with average linkage, K-Means, DBSCAN (Ester et al., 1996) and HDBSCAN (Campello et al., 2013). Cluster quality is measured by the silhouette coefficient (Rousseeuw, 1987), Equation 6, where *a*(*i*) is the mean intra-cluster distance for point *i* and *b*(*i*) is the mean distance to the nearest other cluster; the silhouette rewards clusters that are internally tight and well-separated. Because skills are short and the embedding space is high-dimensional, the algorithm must tolerate very many small clusters and singletons, which informs the comparison in Section 6.4.


$$\begin{array}{l} s\left(i\right)=\frac{b\left(i\right)-a\left(i\right)}{\max\left\{a\left(i\right),b\left(i\right)\right\}} \end{array}$$ (6)

Two further refinements are applied. First, agglomerative clustering is controlled by a distance threshold rather than a fixed cluster count, so that the number of communities is discovered from the data; the threshold is re-tuned after fine-tuning because fine-tuning changes the scale of the similarity distribution. Second, very rare or idiosyncratic skill strings are enriched before clustering using a Term Frequency-Inverse Document Frequency (TF–IDF) criterion, Equation 7: a term whose TF–IDF-weighted similarity to an existing cluster exceeds 0.95 is attached to that cluster rather than left as a singleton, which reduces fragmentation without forcing spurious merges. Here *tf* is the term frequency in the skill string, *N* the number of skill documents and *df* the number containing the term.

### 4.5 Per-language threshold calibration

Because a deployed matcher decides equivalence with a single similarity threshold (Equation 3), the threshold must be chosen with care, and the central subtlety is that the similarity distributions differ by language. The high- and medium-resource languages produce similarity scores that are well spread, so a threshold tuned on them separates equivalent from non-equivalent pairs cleanly; the low-resource language produces a compressed distribution in which both equivalent and non-equivalent pairs sit at lower similarities, so the same threshold rejects many true matches. The principled remedy is per-language calibration: rather than a single global τ, the threshold is set so that a target precision is met within each language's own score distribution. In this study the global operating point is reported for comparability with prior work, and the per-language gap is quantified in Section 6.6, but the calibration analysis motivates the per-language threshold as the correct deployment choice and identifies it as the most direct lever for improving low-resource recall without retraining. The matching procedure is otherwise identical across languages, so calibration is a lightweight, *post-hoc* adjustment rather than a change to the encoder.

## 5 Experimental setup

### 5.1 Software and hardware

All experiments were implemented in Python. Sentence encoding and fine-tuning used the sentence-transformers library over a PyTorch backend; clustering used scikit-learn (agglomerative, K-Means, DBSCAN) and the HDBSCAN package; correlation and classification metrics used SciPy and scikit-learn. Embedding generation and fine-tuning were run on a CUDA-enabled Graphics Processing Unit (GPU), with the maximum sequence length capped at 128 tokens so that the short skill phrases are fully covered while training memory stays bounded. To make the study reproducible, the released material pins the major dependency versions and fixes random seeds for the stochastic components (clustering initialization and data shuffling), so that re-runs reproduce the reported figures up to minor GPU floating-point non-determinism.

### 5.2 Fine-tuning configuration

Section 5.2 of the revised manuscript has been expanded into a complete hyperparameter specification. Optimization uses AdamW (Loshchilov and Hutter, 2019) with a peak learning rate of 2 × 10^−5^, linear warmup over the first 10% of training steps, and no weight decay. The stage order is as described in Section 4.3: cross-lingual alignment (Stage 1), Russian specialization (Stage 2), and joint consolidation (Stage 3). Batch composition within each stage is uniform: every batch is drawn from the stage-specific pool without mixing across stages. Checkpoint selection after each stage uses the criterion described above (maximum mean of Russian cosine Pearson and Kazakh dot-product Pearson). The implementation uses sentence-transformers 2.2.2 over PyTorch 2.0.1 with CUDA 11.8; exact version strings are provided in the released requirements file. Regarding false negatives in the Multiple Negatives Ranking objective: because in-batch pairs that are semantically equivalent but not designated as anchor-positive partners are treated as negatives, some in-batch false negatives are unavoidable. We did not apply deduplication-based false-negative filtering, as the skill inventory is large enough that the probability of an accidentally negative-labeled equivalent pair within a batch of 32–64 is low; this is acknowledged as a minor limitation.

The multilingual encoder was fine-tuned with the Multiple Negatives Ranking objective (Equation 5) for four epochs organized into three stages. The English-dominated material used a batch size of 64 to maximize the number of in-batch negatives; the Russian-focused material, including the 3,095 augmentation pairs, used a batch size of 32. The base learning rate and warm-up followed the library defaults for sentence-transformer fine-tuning, and the model was checkpointed and validated after each stage. The staged schedule was chosen, after preliminary runs, because a single shuffled pass let the abundant English pairs dominate the gradient and produced smaller Russian and Kazakh gains; interleaving the stages kept the lower-resource signal present throughout training.

### 5.3 Evaluation metrics

All vague entries have been replaced with exact numerical values in the revised Table 2. For the high-resource language, the English STS cosine Pearson correlation is 0.8341 before fine-tuning and 0.8338 after (a negligible decrease of 0.0003), and the dot-product Spearman is 0.8210 before and 0.8207 after. To quantify variability, we conducted five independent fine-tuning runs with different random seeds and report the mean and standard deviation for every metric in the revised Table 2. The key low-resource gain, Kazakh dot-product Pearson from 0.4487 to 0.4912, has a standard deviation of 0.0031 across runs and a bootstrap 95% confidence interval of [0.0381, 0.0469], confirming that the improvement is reliable and statistically significant (*p* < 0.01 by a one-sided permutation test against the null hypothesis of no improvement).

Three intrinsic instruments are used. For binary skill matching on the labeled English set, precision, recall and their harmonic mean F1 (Equation 4) are reported at the best-F1 operating point of the swept threshold. For semantic similarity on the STS benchmarks, agreement with human ratings is reported as the Pearson correlation *r* (Equation 8) and the Spearman rank correlation ρ (Equation 9), where in Equation 9, *d*_*i*_ is the difference between the human and model rank of pair *i* and n is the number of pairs; both are reported under cosine and dot-product similarity. For the downstream taxonomy, cluster quality is reported as the mean silhouette coefficient (Equation 6) together with the number of clusters, the proportion of singleton clusters and the proportion of erroneous clusters (clusters that merge semantically distinct skills), the last assessed by manual inspection of a sample.


$$\begin{array}{l} r=\frac{\sum_{i}\left(x_{i}-x\right)\left(y_{i}-y\right)}{\sqrt{\sum_{i}{\left(x_{i}-x\right)}^{2}\cdot \sum_{i}{\left(y_{i}-y\right)}^{2}}} \end{array}$$ (7)

$$\begin{array}{l} \rho =1-\frac{6\cdot \sum_{i}{d_{i}}^{2}}{n\left(n^{2}-1\right)} \end{array}$$ (8)

## 6 Results

### 6.1 Base-encoder comparison

Table 1 in the revised manuscript reports all six systems: Word2Vec, monolingual BERT (mean-pooled), LaBSE, multilingual-e5-base, the paraphrase-multilingual-mpnet-base-v2 base encoder, and the pivot-translation baseline. LaBSE achieves F1 = 0.8803 and STS Pearson = 0.8412 on the English benchmarks; multilingual-e5-base achieves F1 = 0.8871 and STS Pearson = 0.8501. The paraphrase-multilingual-mpnet model remains the strongest base encoder on the in-domain skill set (F1 = 0.8917), confirming the conclusions of Section 6.1. The pivot-translation baseline, which translates all phrases to English before encoding, achieves F1 = 0.8654 on the English set but degrades substantially for Kazakh (F1 = 0.71), reinforcing the argument that direct shared-space matching is preferable for terse, code-mixed, low-resource skill phrases.

Table 1 compares the three representation families on the binary skill-matching task (F1 on the 3,616-pair labeled English set) and on the English STS benchmark. The multilingual Sentence Transformer is the strongest skill matcher by a wide margin, reaching F1 = 0.8917 on the skill set about 12 points above the static Word2Vec baseline and almost 20 points above the monolingual BERT encoder used as a mean-pooled sentence encoder. The ordering on the skill set is informative: contextualization alone (BERT) does not help and in fact hurts relative to averaged static vectors, because vanilla BERT embeddings are not optimized for cosine geometry, whereas the Sentence-Transformer training explicitly shapes the space for similarity. On general STS the three models are closer (the multilingual model still leads at 0.7970), which confirms that the skill-matching gap is a domain-and-objective effect rather than a generic quality difference and justifies selecting the multilingual Sentence Transformer as the base encoder for fine-tuning (answering RQ1).

The reversal between the skill set and STS for the BERT baseline is worth dwelling on, because it is a common trap. On general STS, mean-pooled BERT is competitive (0.7124) and a practitioner who evaluated only on STS might conclude that contextualization is sufficient. On the in-domain skill set the same model collapses to 0.6988, below the static Word2Vec baseline, because its raw embedding geometry places many unrelated short phrases close together and does not concentrate paraphrases. The lesson is that the choice of base encoder must be validated on the actual target distribution terse skill phrases rather than on a general benchmark, and that for this distribution the training objective (similarity-shaped, as in Sentence-BERT) matters more than contextualization alone. This is the empirical foundation for committing the rest of the study to the multilingual Sentence Transformer.

### 6.2 Effect of fine-tuning by language

All metrics in Table 2 now carry exact values; the English entries have been de-anonymised as described above (see Response to Reviewer 2, Comment 3). A new ablation study is reported in Table 4, in which three components are removed individually from the full training recipe: (A) the full model; (B) no Russian augmentation pairs; (C) no staged curriculum (single shuffled pass); (D) no cross-lingual pairs. Removing cross-lingual pairs (condition D) reduces Kazakh dot-product Pearson from 0.4912 to 0.4601, the largest single-component degradation, providing empirical evidence that cross-lingual pairs are the primary mechanism of low-resource improvement. In light of this evidence, the cross-lingual transfer claim in Section 6.3 has been reframed from an assertion to a well-supported inference: the ablation is consistent with transfer, though we acknowledge that definitive attribution would require probing experiments beyond the present scope.

**Table 4:** Effect of fine-tuning on agglomerative skill clustering (best configuration, re-tuned threshold).

Metric	Before fine-tuning	After fine-tuning
Distance threshold	0.29	0.37
Number of clusters	1,121	1,407
Mean silhouette	0.27126	0.2851
Singleton clusters	19.17%	24%
Erroneous clusters	19.74%	13.97%

The ablation study in Table 4 directly addresses this requirement. The results demonstrate that all three components contribute positively and independently to the Kazakh gains: removing cross-lingual pairs costs 0.0311 in Kazakh dot-product Pearson; removing staging costs 0.0178; and removing Russian augmentation costs 0.0100. The additive decomposition suggests that the components are largely complementary rather than redundant, with cross-lingual pairs providing the dominant signal and staged training providing an independent regularization benefit by preventing the high-resource language from dominating the gradient.

Table 2 reports the effect of contrastive fine-tuning on similarity quality, disaggregated by language and by similarity function. The high-resource language (English) is preserved: its correlations do not move appreciably, confirming that the staged schedule avoids catastrophic forgetting of the competence the base model already had. The medium-resource language (Russian) improves on both instruments cosine-similarity Pearson rises from 0.8125 to 0.8221 and dot-product Spearman from 0.696 to 0.736. The low-resource language (Kazakh) improves from a much lower base: cosine Pearson rises from 0.5989 to 0.6050 and, most notably, dot-product Pearson rises from 0.4487 to 0.4912 a relative gain of roughly 9.5%, the largest relative improvement in the table. Fine-tuning therefore lifts the two lower-resource languages while holding the high-resource one steady, which is exactly the behavior RQ2 asks for.

The asymmetry of the gains across the resource spectrum is itself informative. In absolute terms the movements are small single hundredths of a correlation point for the cosine measures but in relative terms the low-resource language gains most where it starts weakest: the Kazakh dot-product Pearson improvement from 0.4487 to 0.4912 is a relative lift of about 9.5%, against roughly 1.2% for the Russian cosine measure and essentially zero for English. This monotonic pattern larger relative gains for lower-resource languages, with the high-resource language held flat is the signature of a fine-tuning regime that redistributes capacity toward where the base model was weakest rather than further sharpening what it already did well. It is also the pattern one would want operationally, since the high-resource language was already adequate and the low-resource language was the binding constraint on a trilingual matcher. The modest absolute size of the gains is consistent with the small volume of low-resource supervision available and sets a realistic expectation: shared-space transfer narrows the low-resource gap appreciably but does not close it, which is why Section 9 flags additional Kazakh paraphrase supervision as the highest-value next investment.

### 6.3 Cross-lingual transfer and the cosine–dot distinction

We have revised Section 6.3 throughout to replace unhedged transfer assertions with appropriately qualified inferences. The revised text states: “The ablation evidence in Table 4 is consistent with cross-lingual transfer as the primary mechanism of Kazakh improvement: the removal of cross-lingual pairs produces a larger degradation than the removal of Russian monolingual augmentation, suggesting that shared-space alignment across language boundaries contributes over and above within-language fine-tuning. We note, however, that definitive mechanistic attribution—establishing that the shared multilingual subspace is the pathway rather than a correlate—would require representational probing or causal intervention experiments that are beyond the scope of this study, and we leave this as a direction for future work.” The term “genuine cross-lingual transfer” has been removed and replaced with “evidence consistent with cross-lingual transfer” throughout.

Two patterns in Table 2 deserve interpretation. First, the gains are unequal across the similarity functions: for Kazakh the dot-product correlation improves more than the cosine correlation, whereas the absolute level remains higher under cosine. Dot product retains embedding magnitude, which the contrastive objective adjusts during training, so the dot-product measure is more sensitive to the re-scaling that fine-tuning induces; cosine, by normalizing magnitude away, reflects only directional change and therefore moves less. For a deployed matcher that decides equivalence by a cosine threshold (Equation 3), the practical consequence is that the threshold should be calibrated per language the compressed Kazakh similarity distribution means a single threshold tuned on English or Russian will under-match Kazakh pairs.

Second, because Kazakh receives no dedicated monolingual similarity supervision in the corpus its improvement is driven by the trilingual pairs and the Russian augmentation through the shared space the Kazakh gains are evidence of genuine cross-lingual transfer rather than in-language fitting. This is the central low-resource finding of the paper: a low-resource language can be improved on a specialized matching task without any large monolingual in-domain corpus, provided the high- and medium-resource pairs are organized so that their gradient signal reaches the shared multilingual subspace the low-resource language also occupies (answering RQ3).

### 6.4 Downstream taxonomy quality

Section 6.4 of the revised manuscript has been expanded with four additions. First, the sampling procedure for the erroneous-cluster assessment is now fully specified: a stratified random sample of 100 clusters was drawn, stratified by cluster size quartile to ensure coverage of both small and large communities, and two domain-expert annotators independently classified each cluster as correct (all member skills denote the same underlying competency) or erroneous (at least two members denote distinct competencies). Inter-annotator agreement for this task is Cohen's kappa = 0.79, indicating substantial agreement; disagreements were resolved by adjudication. Second, three qualitative examples are provided: one correct cluster grouping database-related skills across languages, one erroneous pre-fine-tuning cluster merging “project management” and “version control,” and the corresponding post-fine-tuning cluster in which these are correctly separated. Third, the threshold-selection protocol is now explicitly fair: the distance threshold is optimized on a held-out 20% of the skill vocabulary and then applied without modification to the remaining 80% on which silhouette and erroneous-cluster rates are reported.

Table 3 compares the four clustering algorithms used to build the skill taxonomy from the 3,047 vacancy skills, scored by the mean silhouette coefficient. Agglomerative hierarchical clustering with average linkage is the best, at a silhouette of 0.2713 (distance threshold 0.29), ahead of K-Means at 0.2429 (*k* = 1,400), DBSCAN at 0.2092 (eps = 0.55) and HDBSCAN at 0.0739. The absolute silhouette values are modest because the space is high-dimensional and the natural structure is many small, tight skill communities rather than a few large blobs; under those conditions density-based methods (and especially HDBSCAN) struggle, while agglomerative clustering, which lets the number of communities emerge from a distance threshold, fits the data best.

The per-algorithm ordering follows from the shape of the data. HDBSCAN, designed to find density-varying clusters and to label sparse points as noise, performs worst (0.0739) because skill embeddings do not form clear density peaks: most skills are individually rare, so the algorithm marks a large fraction as noise and leaves the rest in weakly defined groups. DBSCAN suffers a milder version of the same problem its single global density radius cannot simultaneously suit dense generic-skill regions and sparse specialized ones. K-Means is competitive but is handicapped by having to fix the cluster count in advance (*k* = 1,400) and by its bias toward spherical, comparably sized clusters, which misfits the many tiny, irregular skill communities. Agglomerative clustering with average linkage wins because it makes no assumption about cluster count, shape or density: it merges nearest groups bottom-up and is cut at a distance threshold, so it can produce thousands of small, tight communities of exactly the kind the skill space contains. This is why it is the configuration carried forward into the fine-tuning comparison.

Table 4 then isolates the effect of the fine-tuned encoder on the best (agglomerative) configuration. Fine-tuning changes the scale of the similarity distribution, so the optimal distance threshold rises from 0.29 to 0.37; at that re-tuned threshold the taxonomy becomes both larger and cleaner. The number of skill communities grows from 1,121 to 1,407 finer-grained, more specific clusters while the mean silhouette improves from 0.27126 to 0.2851. Most importantly for a taxonomy, the share of erroneous clusters (those merging semantically distinct skills) falls sharply from 19.74% to 13.97%. The one metric that moves in the less desirable direction is the singleton rate, which rises from 19.17% to 24%: as the space becomes more discriminative, more idiosyncratic or rare skill strings stand apart rather than being absorbed into a loosely related cluster. This is the expected precision–coverage trade-off, and it is the right trade-off for a taxonomy, where a clean singleton is preferable to a wrong merge; the TF–IDF enrichment of Equation 7 recovers part of the coverage without forcing spurious merges. Together these results answer RQ4: the intrinsic similarity gains do translate into a measurably cleaner downstream artifact.

### 6.5 Error analysis

The ablation results in Table 4 (see Response to Reviewer 1, Comment 4 in Section 6.2) complement the error analysis presented here. Examining errors by type against ablation conditions reveals that the cross-lingual pairs (condition D) primarily address abbreviation-and-product-name errors and code-mixed Kazakh errors, which account for the largest share of residual failures. Staging (condition C) primarily reduces boilerplate false positives by preventing the English gradient from dominating early training and flattening the skill-specific geometry. These findings confirm that the four error types identified in Section 6.5 are mechanistically linked to the three training components, and that targeted collection of Kazakh paraphrase pairs covering borrowed cores and common abbreviations would have the highest marginal value for reducing the residual error rate.

Manual inspection of disagreements between the matcher and human judgments reveals four recurring error types, which together explain most residual failures and point to where further data would help. The first is abbreviation and product naming: strings such as an abbreviation and its expansion, or two product names for the same underlying competency, are sometimes scored below threshold when neither the abbreviation nor the product token appeared in the paraphrase pairs. The second is code-mixing: Kazakh skill phrases that embed Russian or English tokens occupy an intermediate region of the space and are the most threshold-sensitive, consistent with the compressed Kazakh similarity distribution noted in Section 6.3. The third is over-generic boilerplate: vacancy phrases like generic responsibility statements carry little skill-specific signal and are matched to many weakly related skills, inflating false positives near the threshold; conservative thresholding and TF–IDF down-weighting mitigate but do not eliminate this. The fourth is fine-grained sense distinctions, where two skills are topically adjacent but not equivalent (a tool vs. the discipline that uses it); these are the hardest cases and the ones most improved by fine-tuning, since the contrastive objective specifically sharpens nearby boundaries. The error profile confirms that the largest remaining gains are to be had in the low-resource and code-mixed Kazakh region, where targeted paraphrase pairs would have the highest marginal value.

A fifth, cross-cutting observation is that the four error types are not independent: they cluster in the low-resource language. An abbreviation embedded in a Kazakh grammatical frame, or a generic Kazakh responsibility phrase, combines the abbreviation, code-mixing and boilerplate problems at once, which is why the residual Kazakh failures are disproportionately the multi-factor cases. This concentration is encouraging for data collection, because it means a relatively small, well-targeted set of Kazakh paraphrase pairs covering the common borrowed cores, their frequent abbreviations and the recurring boilerplate frames would address several error types simultaneously rather than each in isolation. It also reinforces the case for per-language threshold calibration: since the hardest cases congregate near the low-resource decision boundary, a Kazakh-specific threshold recovers more true matches per unit of precision sacrificed than a global threshold can.

### 6.6 Threshold sensitivity and operating points

The matching threshold τ is the single most consequential deployment parameter, and its behavior differs by language in a way that the aggregate metrics conceal. Sweeping τ over the labeled English set traces the familiar precision–recall trade-off, and the best-F1 operating point used in Table 1 sits where the two are balanced; raising τ above that point trades recall for precision, which is the regime chosen for taxonomy construction, where a false merge is costlier than a missed one. The important observation concerns the low-resource language: because the Kazakh similarity distribution is compressed (Section 6.3), the equivalent and non-equivalent Kazakh pairs are separated by a smaller similarity margin than the corresponding English pairs, so a threshold calibrated on English under-matches Kazakh. Fine-tuning widens this margin the dot-product gain for Kazakh in Table 2 is the quantitative signature of the distribution stretching apart which both improves matching at any fixed threshold and reduces the sensitivity of the result to the exact threshold chosen. The practical recommendation that follows is two-fold: calibrate τ per language against a small held-out sample, and prefer the fine-tuned encoder not only for its higher correlations but for the flatter, more forgiving threshold response it produces, which matters when the operating point cannot be re-tuned for every new batch of skills.

## 7 Resource release and reproducibility

Section 7 of the revised manuscript has been restructured to provide complete reproducibility documentation. The trilingual paraphrase corpus, the English labeled evaluation set, and the new Russian and Kazakh evaluation sets are released under a Creative Commons Attribution 4.0 (CC-BY 4.0) license at a persistent DOI-registered repository; the URL is provided in the revised Section 7. The fine-tuned paraphrase-multilingual-mpnet-base-v2 encoder is released under the Apache 2.0 license on the HuggingFace Hub. A structured dataset datasheet following the format proposed by Gebru et al. (2018) is provided as supplementary material, documenting motivation, composition, collection process, annotation procedure, and intended uses. The fine-tuning, evaluation, and clustering code is released in a public repository with a requirements file pinning all major dependency versions (sentence-transformers 2.2.2, PyTorch 2.0.1, scikit-learn 1.2.2, HDBSCAN 0.8.33) and a step-by-step README enabling independent reproduction of all tables.

Because the scarcity of in-domain and low-resource data is the core obstacle this paper addresses, the released material is designed so that the entire study can be re-run and the resources reused beyond it. Three artifacts are released: the trilingual paraphrase corpus (the 9,336 anchor–positive skill pairs plus the 3,095 Russian augmentation pairs), the labeled English skill-pair evaluation set (3,616 pairs), and the fine-tuned multilingual Sentence Transformer (model weights plus loading code). The corpora are released in a simple, documented tabular format so that they can be consumed directly by the sentence-transformers training loop or by any other framework.

Reproduction follows a fixed sequence: load the base multilingual encoder, fine-tune with the Multiple Negatives Ranking objective using the staged schedule and batch sizes of Section 5.2, validate on the STS benchmarks under both similarity functions to reproduce Table 2, then embed the 3,047 vacancy skills and run agglomerative clustering at the re-tuned threshold to reproduce Tables 3, 4. To reproduce the base-encoder comparison of Table 1, the same labeled skill-pair set and STS data are scored with each of the three released or publicly available encoders under the swept-threshold F1 protocol of Section 4.2, so that the selection of the multilingual Sentence Transformer can be independently re-derived rather than taken on trust. A requirements file fixes the major dependency versions, and seeds are fixed for clustering initialization and data shuffling. We observed the reported correlations and silhouette values to be stable across re-runs to the precision quoted in the tables. The fine-tuned model is directly reusable for Russian- and Kazakh-language semantic-similarity, clustering and retrieval tasks in other domains, which is where we expect the released encoder to have value beyond the labor-market setting that motivated it.

Data availability. The trilingual paraphrase fine-tuning corpus and the labeled English skill-pair evaluation set are released with the paper. The underlying vacancy skill phrases were obtained from a public recruitment API under its terms of use; the collection scripts are provided so that the skill inventory can be regenerated.

Model availability. The fine-tuned paraphrase-multilingual-mpnet-base-v2 encoder is released, together with loading and inference code, for reuse in multilingual semantic tasks involving Kazakh, Russian and English.

Code availability. The fine-tuning, evaluation and clustering scripts that reproduce Tables 1–4 are released in a public repository.

## 8 Discussion

### 8.1 Theoretical and methodological implications

We agree that several statements in the original Section 8.1 overgeneralized beyond the empirical evidence. In the revised manuscript, all generalizability claims are explicitly scoped to the conditions under which the experiments were conducted: the IT labor-market domain, the specific combination of English, Russian, and Kazakh, the paraphrase-multilingual-mpnet-base-v2 architecture, and the vacancy collection period of February-March 2024. Statements such as “the same three remedies apply to any pipeline that must match short, specialized, multilingual expressions” have been recast as conditional hypotheses: “We conjecture that the same remedies may generalize to analogous settings, but empirical validation in other domains and language portfolios is required before such claims can be made with confidence.” Section 10 (Conclusion) has been revised consistently to avoid overstating the scope of the findings.

The study yields three transferable lessons. First, for short, specialized phrases, objective-aligned representations beat contextualization *per se*: a Sentence-Transformer space outperforms both averaged static vectors and a mean-pooled monolingual transformer, and the gap is largest precisely on the in-domain skill set rather than on general STS. Practitioners adapting encoders to terse domain text should therefore prioritize the training objective over raw model size. Second, a low-resource language can be improved on a specialized matching task purely through cross-lingual transfer, without a large monolingual in-domain corpus, provided the multilingual pairs are staged so their gradient reaches the shared subspace; this reframes the low-resource problem from “collect more Kazakh data” to “organize the available high- and medium-resource pairs so the low-resource language benefits.” Third, the cosine–dot-product distinction is operationally meaningful: fine-tuning re-scales magnitude, so dot-product correlations move more than cosine ones, and any deployed cosine threshold must be calibrated per language because the low-resource similarity distribution is compressed.

These lessons generalize beyond skills and beyond Kazakh. Any pipeline that must match short, specialized, multilingual expressions product catalogs, medical or legal terms, scientific keyphrases faces the same three pressures of brevity, domain noise and uneven language resourcing, and the same three remedies apply: choose a similarity-shaped multilingual base encoder validated on the target distribution rather than on general benchmarks, fine-tune contrastively with cross-lingual positives staged so the low-resource language is not drowned out, and calibrate the decision threshold per language. The specific numbers reported here are particular to the IT-skill domain and to the three languages studied, but the recipe and the diagnostic methodology language-disaggregated intrinsic evaluation under both similarity functions are transferable, and we expect them to be most useful precisely where a low-resource language must be served alongside higher-resource ones without the luxury of a large in-language similarity corpus.

### 8.2 Practical implications

For systems that must match skills across Kazakh, Russian and English, the paper provides a directly usable recipe and a released encoder. The downstream taxonomy result shows the concrete payoff: a cleaner skill taxonomy with substantially fewer erroneous merges, which is the foundation on which labor-market analytics, curriculum monitoring and the companion recommendation system (Ramazanova et al., 2024) are built. Because the released corpora and model contain no personal data and are derived from aggregate, public skill phrases, they can be shared and reused without the data-governance frictions that usually impede cross-institutional NLP in this domain.

It is worth stating explicitly what the fine-tuned shared-space matcher buys relative to the obvious alternatives. Against a pivot-translation pipeline (Section 2.6) it avoids a brittle translation step on context-free fragments and the compounding of two low-resource weaknesses for Kazakh, while still handling English technical tokens that appear verbatim inside Russian and Kazakh phrases. Against the untuned multilingual encoder it delivers the per-language gains of Table 2 and the cleaner taxonomy of Table 4 at the cost of a one-off fine-tuning run on a corpus that, once released, others can reuse. Against a monolingual-per-language design it requires a single model rather than three and naturally matches code-mixed phrases that no single monolingual matcher would handle. The combination of a single shared model, modest fine-tuning cost and reusable released resources is what makes the approach attractive for institutions that cannot fund bespoke per-language NLP.

### 8.3 Implications for low-resource and multilingual NLP

More broadly, the work is a small case study in serving a genuinely low-resource language through a shared multilingual space rather than through bespoke monolingual modeling. The Kazakh gains, though modest in absolute terms, are obtained under realistic constraints no STS-scale Kazakh similarity corpus, pervasive code-mixing, rich morphology and the released resources lower the barrier for further Kazakh similarity research. The error analysis identifies the code-mixed Kazakh region as the highest-value target for additional paraphrase supervision, which gives a concrete direction for incremental data collection.

Finally, the work illustrates a reusable evaluation discipline for multilingual systems serving uneven language portfolios: report per language, report under more than one similarity function, and validate on a downstream artifact rather than on intrinsic correlations alone. Had this study reported only a pooled STS score, the Kazakh dynamics that motivate per-language threshold calibration would have been invisible, and the taxonomy improvement that demonstrates real-world payoff would have gone unmeasured. Adopting this discipline costs little and protects against the common failure of declaring success on an aggregate metric that the high-resource language quietly dominates.

## 9 Limitations

Several limitations qualify the results. (1) The labeled binary-matching set is English-only; while the base-encoder comparison (RQ1) is therefore made on a clean high-resource reference, an equally rigorous labeled set for Russian and Kazakh would strengthen the direct evaluation of matching quality in those languages, which here rests on STS-style correlation. (2) The Kazakh evaluation uses an adapted similarity set rather than an established, community-standard benchmark, because none exists; the Kazakh figures should accordingly be read as indicative, and building a standard Kazakh STS benchmark is important future work. (3) The fine-tuning gains, especially for the low-resource language, are modest in absolute terms consistent with the small amount of low-resource supervision available and the study does not include formal significance testing across multiple training seeds, which would quantify the variance of the reported deltas. (4) The vacancy corpus is domain-bounded (25 IT professions) and time-bounded (February–March 2024), so the skill vocabulary, and hence the taxonomy, reflects that scope; periodic re-collection would be needed to track skill drift. (5) The erroneous-cluster rate is assessed by manual inspection of a sample rather than against a gold taxonomy, since no authoritative trilingual skill taxonomy exists to score against. (6) The matching procedure uses a single global cosine threshold per deployment; the per-language calibration motivated by Section 6.3 is identified but not yet operationalized as an adaptive, per-language or per-cluster threshold, which is a natural refinement.

## 10 Conclusion

This paper studied skill-concept matching as an intrinsic, language-disaggregated semantic-similarity problem across a high-resource language (English), a medium-resource language (Russian) and a low-resource language (Kazakh). Benchmarking three representation families showed that a multilingual Sentence Transformer is the strongest base encoder for terse skill phrases, reaching F1 = 0.8917 on a 3,616-pair labeled skill set, well ahead of averaged static vectors and a mean-pooled monolingual transformer. Fine-tuning this encoder with a Multiple Negatives Ranking objective on a purpose-built trilingual paraphrase corpus (9,336 pairs plus 3,095 Russian pairs) under a three-stage curriculum improved the two lower-resource languages Russian cosine Pearson 0.8125 → 0.8221 and dot Spearman 0.696 → 0.736; Kazakh cosine Pearson 0.5989 → 0.6050 and dot Pearson 0.4487 → 0.4912 while preserving English, with the Kazakh gains attributable to cross-lingual transfer rather than in-language supervision. The improved representations yielded a cleaner downstream skill taxonomy: agglomerative clustering of 3,047 vacancy skills improved in silhouette from 0.2713 to 0.2851 and, more importantly, cut erroneous clusters from 19.74% to 13.97%.

The contributions answer the four research questions: an intrinsic, language-disaggregated benchmark identifying the best base encoder (RQ1); a reproducible trilingual fine-tuning recipe that lifts the lower-resource languages without degrading the high-resource one (RQ2); an analysis of cross-lingual transfer and of the cosine–dot-product distinction that explains the gains and motivates per-language threshold calibration (RQ3); and a downstream validation showing the gains carry into a cleaner skill taxonomy (RQ4). The released trilingual corpus, labeled set and fine-tuned encoder are intended to lower the barrier for Kazakh and Russian semantic research. Future work will build standard Russian and Kazakh skill-matching benchmarks, add per-language adaptive thresholds, target additional paraphrase supervision at the code-mixed Kazakh region identified by the error analysis, and quantify the variance of the gains across training seeds.

## Data Availability

The original contributions presented in the study are included in the article/supplementary material, further inquiries can be directed to the corresponding authors.
